# Association between nirmatrelvir/ritonavir treatment and antibiotic prescribing in the outpatient setting among patients with COVID-19

**DOI:** 10.1128/spectrum.03209-24

**Published:** 2025-03-05

**Authors:** Aisling R. Caffrey, Haley J. Appaneal, Vrishali V. Lopes, Thomas Lavoie, Laura Puzniak, Evan J. Zasowski, Luis Jodar, Iqra Arham, Kerry L. LaPlante, John M. McLaughlin

**Affiliations:** 1 Infectious Diseases Research Program, Providence Veterans Affairs Medical Center, Providence, Rhode Island, USA; 2 Center of Innovation in Long-Term Support Services, Providence Veterans Affairs Medical Center, Providence, Rhode Island, USA; 3 College of Pharmacy, University of Rhode Island, Kingston, Rhode Island, USA; 4 School of Public Health, Brown University, Providence, Rhode Island, USA; 5 Pfizer Inc., New York, New York, USA; University of Texas Southwestern Medical Center, Dallas, Texas, USA

**Keywords:** COVID-19, nirmatrelvir/ritonavir, antibiotics, stewardship, co-infection

## Abstract

Coronavirus disease 2019 (COVID-19) has complicated the management of acute respiratory infections and impacted antibiotic use. We assessed the relationship between nirmatrelvir/ritonavir (NMV/r) receipt and outpatient antibiotic prescribing among patients with COVID-19 in a large national health system. We conducted a retrospective cohort study among outpatients enrolled in the Veterans Affairs Healthcare System who had a positive severe acute respiratory syndrome coronavirus 2 test or COVID-19 diagnosis and were eligible for NMV/r treatment between 1 April 2022 and 31 March 2024. NMV/r-treated patients were compared with those who did not receive NMV/r and were considered unexposed until NMV/r was dispensed. We assessed the relationship between NMV/r receipt and being prescribed an outpatient antibiotic in the 30 days after a COVID-19 diagnosis using adjusted Cox proportional hazards regression. We included 302,600 NMV/r-eligible outpatients with COVID-19, of whom 67,649 received NMV/r and 234,951 did not receive NMV/r. NMV/r-treated patients were less likely to receive outpatient antibiotics compared to those who did not receive NMV/r (7.2% [4,901/67,649] vs 9.2% [21,533/234,951], respectively; adjusted hazard ratio [HR] 0.65, 95% CI: 0.63‒0.68). After excluding patients who received an antibiotic prescription upon COVID-19 diagnosis (i.e., likely empiric therapy), this relationship was attenuated (HR: 0.91, 95% CI: 0.87‒0.95). NMV/r-eligible patients with COVID-19 who received NMV/r were 35% less likely to be prescribed outpatient antibiotics compared to patients who did not receive NMV/r, possibly driven by a diminished perceived need for empiric antibiotic therapy. Treatment with NMV/r may reduce unnecessary outpatient antibiotic use. Antibiotics should be reserved for patients with a high suspicion of bacterial co-infection.

**IMPORTANCE:**

Antimicrobial resistance, driven by the overuse of antibiotics, is a major global health threat. The coronavirus disease 2019 (COVID-19) pandemic has complicated this issue, with antibiotics often prescribed to patients with COVID-19 despite being ineffective against viruses. These practices, typically aimed at preventing or empirically treating rare bacterial co-infections, have raised concerns about accelerating resistance. The antiviral nirmatrelvir/ritonavir (NMV/r), widely used in high-risk patients with COVID-19 to prevent severe illness, offers an opportunity to reassess antibiotic use in patients with respiratory infections. Our study of over 300,000 patients in a national healthcare system found that those treated with NMV/r for COVID-19 were 35% less likely to receive antibiotics than those who did not receive the antiviral. Lower antibiotic use among patients treated with NMV/r may reflect a reduction in unnecessary outpatient antibiotic use. These findings highlight the potential role of antivirals in supporting antibiotic stewardship and addressing a critical public health challenge.

## INTRODUCTION

The overuse and misuse of antibiotics have led to antimicrobial resistance, which has been identified by the World Health Organization as one of the world’s top 10 public health threats. The coronavirus disease 2019 (COVID-19) pandemic has compounded the issue of antibiotic misuse. While antibiotics do not affect viruses, they have been widely prescribed to patients with COVID-19, often empirically to prevent or treat secondary bacterial infections (1–3). Several recent studies, however, have shown that bacterial co-infection in patients with COVID-19 is relatively uncommon (i.e., likely <5%) (2, 4–6). These findings have raised concerns about accelerating the development of antibiotic-resistant bacteria and the unnecessary use of antibiotics among patients with COVID-19 (7).

The introduction of severe acute respiratory syndrome coronavirus 2 (SARS-CoV-2) antiviral treatments has provided an opportunity to reassess antibiotic use in the context of COVID-19. Nirmatrelvir/ritonavir (NMV/r), an oral SARS-CoV-2 antiviral given in the outpatient setting, has been shown to be effective for treating COVID-19 in those at high risk for progression to severe disease (8–11). NMV/r has been used widely in the United States since it was authorized for emergency use in December 2021 for individuals 12 years of age and older, weighing at least 40 kg, who are at high risk for progression to severe COVID-19. NMV/r was subsequently fully approved by the U.S. Food and Drug Administration on 25 May 2023 for high-risk adults. The ability of NMV/r to prevent progression to severe COVID-19 could potentially lead to a decrease in the perceived need for antibiotics among patients with COVID-19. This, in turn, could have important implications for antibiotic stewardship programs and strategies to combat antimicrobial resistance. Moreover, SARS-CoV-2 antiviral treatment may also, in rarer instances, reduce secondary infection. We evaluated the impact of NMV/r on outpatient antibiotic prescribing patterns among NMV/r-eligible patients with COVID-19.

## MATERIALS AND METHODS

### Design, setting, and population

This retrospective cohort study included adults ≥18 years of age who were enrolled in the Veterans Affairs (VA) Healthcare System and who tested positive for SARS-CoV-2 (via PCR or antigen test) or were diagnosed with COVID-19 (International Classification of Diseases, Tenth Revision, Clinical Modification [ICD-10] code U07.1) between 1 April 2022 and 31 March 2024 in the outpatient setting of the VA Healthcare System. The index date was defined as the date of the earliest positive SARS-CoV-2 test or COVID-19 diagnosis. Only the first COVID-19 episode for each patient was included in the study. To be eligible, participants had to (i) have at least one VA Healthcare System visit in the preceding 12 months (i.e., an “active” VA user) and (ii) meet Centers for Disease Control and Prevention (CDC) criteria for NMV/r eligibility (i.e., at high risk for progression to severe COVID-19), which was defined as being ≥50 years of age, having a CDC-defined high-risk medical condition, or not being up-to-date with COVID-19 vaccinations (12). We excluded patients (i) with severe renal impairment (estimated glomerular filtration rate [eGFR] <30 mL/min in the previous 180 days), (ii) with moderate or severe liver disease, (iii) who were currently taking medications contraindicated for use with NMV/r (Table S1), (iv) who were treated with molnupiravir, remdesivir, or any SARS-CoV-2 monoclonal antibodies (i.e., bamlanivimab, bebtelovimab, casirivimab/imdevimab, cilgavimab/tixagevimab, and sotrovimab) in the 30 days prior to the index date (13–16), (v) who received <5 days supply of NMV/r, (vi) who had previously received NMV/r in the 30 days prior to the index date, or (vii) who received NMV/r ≥ 6 days after the index date.

Patient data were extracted from clinical records in the Veterans Health Administration Corporate Data Warehouse, a comprehensive repository of health data within the national VA Healthcare System. The database encompasses a wide range of information including demographic details, records of inpatient and outpatient encounters (including procedure codes), pharmacy data (including medication dispensing and administration details as well as records of medications obtained outside the VA system), ICD-10 diagnosis codes, laboratory and microbiology records, vital statistics, and other health-related data. This study was determined to be exempt by the VA Providence Healthcare System (VAPHS) Institutional Review Board (IRB) and approved by the VAPHS Research and Development Committee. As this was a retrospective study of existing health records and exempt from IRB review, informed consent requirements were not applicable.

### Exposure

The exposure of interest was the receipt of a 5-day supply of NMV/r (defined using outpatient pharmacy dispensing data) within 5 days of the index date compared to not receiving NMV/r. Patients were considered unexposed until the date that NMV/r was dispensed.

### Outcomes

The outcome of interest was the receipt of an outpatient antibiotic prescription in the 30 days following the index date (i.e., the date of earliest positive SARS-CoV-2 test or COVID-19 diagnosis) (17). Antibiotics were identified from dispensed outpatient prescriptions. Only outpatient antibiotics commonly used to treat respiratory tract infections were assessed (Table S2). Patients were censored if they received other COVID-19 antiviral treatment after the index date (e.g., molnupiravir, remdesivir, or SARS-CoV-2 monoclonal antibodies), were hospitalized, or died during the 30-day follow-up period.

### Statistical analysis

Prior literature and subject matter expertise were used to select variables that could potentially confound the relationship between NMV/r treatment and antibiotic use in the outpatient setting. NMV/r exposure was modeled as time varying (i.e., participants were considered unexposed until the date of NMV/r dispense). Cox proportional hazards models were adjusted for the following covariates: week of SARS-CoV-2 infection or COVID-19 diagnosis, whether an outpatient visit occurred at the time of testing positive or COVID-19 diagnosis (yes/no) (8), age group (18‒49, 50‒64, 65‒74, 75‒84, and ≥85 years), sex (male or female), race (Black or African American, White, and other), ethnicity (Hispanic or Latino and not Hispanic or Latino), region (Midwest, Northeast, West, and South), socioeconomic indicators (measured by the area deprivation index [ADI] grouped into quintiles from least to most deprived) (18), body mass index category (underweight [<18.5], normal weight [18.5‒24.9], overweight [25.0‒29.9], obese [≥30.0], or missing) (19), Charlson comorbidity index (0, 1, 2, 3, and ≥4), history of medical conditions (yes/no; modeled individually), including cancer, asthma, chronic obstructive pulmonary disease, hypertension, congestive heart failure, atherosclerosis, or other heart disease, cognitive disorders, including dementia, diabetes, HIV/AIDS, or liver disease, history of immunocompromising conditions, or use of immunosuppressive treatment (yes/no) (20), smoking status (current or former, never, and unknown), prior healthcare interactions (primary care visit in the previous year [yes/no], hospitalization in the previous year [yes/no], and emergency department or urgent care visit in the previous year [yes/no]), use of medications with the potential for drug-drug interactions with NMV/r in the 90 days prior to index (use an alternate COVID-19 therapy, hold drug, dose adjust drug, monitor drug, or not on concomitant medication with drug-drug interactions [DDI] potential) (16, 21), antibiotic use in the 30 days prior to index (yes/no), COVID-19 vaccination status (up-to-date [defined as receiving at least one dose of XBB vaccine if the index date occurred between 25 September 2023 and 31 March 2024, at least one dose of a BA.4/5 bivalent vaccine if the index date occurred between 1 September 2022 and 24 September 2023, or at least three doses of wild-type COVID-19 vaccine if the index date occurred between 1 April 2022 and 31 August 2022], previously vaccinated against COVID-19 but not up-to-date, and never received COVID-19 vaccine), receipt of influenza vaccine in the previous year (yes/no), receipt of pneumococcal vaccine in the previous 5 years (yes/no), prior documented SARS-CoV-2 infection (yes/no), current (index date to 7 days post index) urinary tract infection (time varying), history of urinary tract infection in the 30 days prior to index (yes/no), and history of acute respiratory infection in the 30 days prior to index (yes/no).

The underlying Cox model assumption of proportional hazards was verified through formal testing (Schoenfeld residuals) and graphical analysis. In sensitivity analyses, we evaluated the impact of excluding patients who received an antibiotic prescription on the index date (i.e., likely empiric therapy). Additional sensitivity analyses were also performed using propensity score matching (nearest neighbor) and only including patients with positive SARS-CoV-2 lab tests. All statistical analyses were carried out using SAS software (Version 9.4 and Enterprise Guide 8.3, SAS Institute Inc., Cary, NC, USA).

## RESULTS

**Fig 1:**
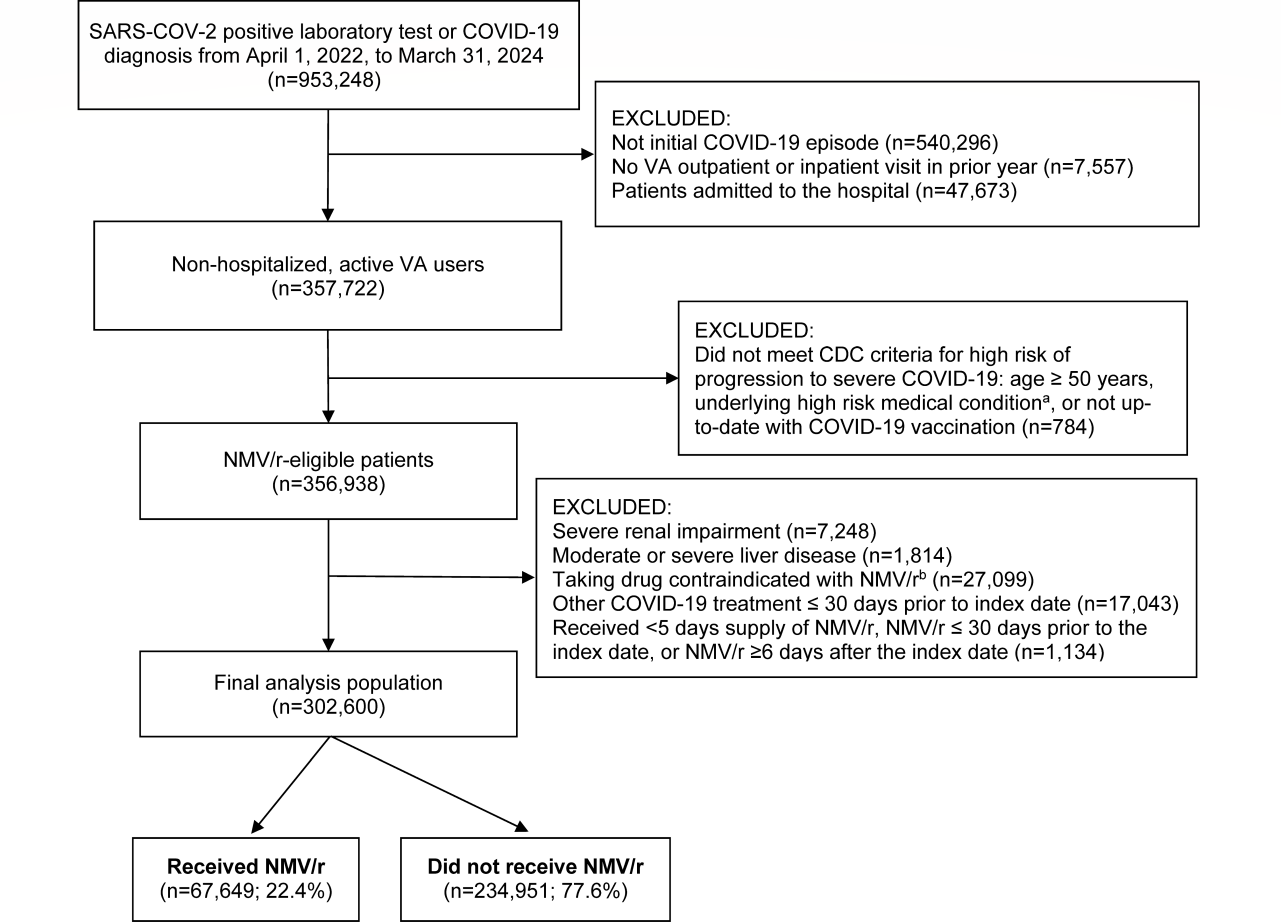
Study selection criteria. ^a^Asthma, cancer, cardiomyopathies, cerebrovascular disease, chronic heart failure, chronic kidney disease, chronic liver disease, chronic lung disease, coronary artery disease, current or former smoker, cystic fibrosis, delirium, dementia, and other cognitive disorders, diabetes mellitus type 1 or 2, HIV, immunocompromised, mental health conditions, obesity, pregnancy, and tuberculosis. ^b^Drugs contraindicated with NMV/r that require the use of an alternative COVID-19 therapy, with any days supply during the 90 days prior to the index date.

Our study included 67,649 NMV/r-treated and 234,951 NMV/r-untreated outpatients within the VA Healthcare System who tested positive for SARS-CoV-2 or were diagnosed with COVID-19 and met study inclusion criteria (Fig. 1). Patients who received NMV/r (compared to those who did not receive NMV/r) were more likely to be ≥65 years of age (53.3% vs 46.9%, respectively, *P* < 0.001), be obese (53.1% vs 49.8%, *P* < 0.001), have a Charlson comorbidity score ≥1 (58.2% vs 53.2%, *P* < 0.001), be up-to-date with COVID-19 vaccination (37.2% vs 30.9%, *P* < 0.001), have received an influenza vaccine in the prior year (61.2% vs 53.4%, *P* < 0.001), and have received a pneumococcal vaccine in the prior 5 years (39.5% vs 34.3%, *P* < 0.001). Table 1 describes patient characteristics by receipt of NMV/r. Among patients who received NMV/r, treatment was most often initiated on the index date (55,537/67,649; 82.1%) or on the day after index (9,946/67,649; 14.7%).

**TABLE 1:** Patient characteristics by receipt of NMV/r^*f*^

	Received NMV/r (*n* = 67,649)	Did not receive NMV/r (*n* = 234,951)	*P* value
Received outpatient antibiotic in 30 days following a positive SARS-CoV-2 test or COVID-19 diagnosis			<0.001
Yes	4,901 (7.2)	21,533 (9.2)	
No	62,748 (92.8)	213,418 (90.8)	
Variant time period			<0.001
Pre-XBB Omicron	36,626 (54.1)	153,718 (65.4)	
XBB	23,567 (34.8)	61,933 (26.4)	
JN.1	7,456 (11.0)	19,300 (8.2)	
Age group			<0.001
18–49 years	11,570 (17.1)	57,778 (24.6)	
50–64 years	19,963 (29.5)	67,144 (28.6)	
65–74 years	19,362 (28.6)	57,102 (24.3)	
75–84 years	13,749 (20.3)	41,740 (17.8)	
>85 years	3,005 (4.4)	11,187 (4.8)	
Sex			<0.001
Male	57,944 (85.7)	199,807 (85.0)	
Female	9,705 (14.3)	35,144 (15.0)	
Race			<0.001
Black or African American	14,132 (20.9)	50,506 (21.5)	
White	45,943 (67.9)	155,883 (66.3)	
Other race	7,574 (11.2)	28,562 (12.2)	
Ethnicity			<0.001
Hispanic or Latino	6,013 (8.9)	24,925 (10.6)	
Not Hispanic or Latino	61,636 (91.1)	210,026 (89.4)	
Region			<0.001
Midwest	15,673 (23.2)	44,449 (18.9)	
Northeast	10,756 (15.9)	35,746 (15.2)	
West	16,414 (24.3)	53,497 (22.8)	
South	24,806 (36.7)	101,259 (43.1)	
ADI (18) Quintile			<0.001
1 (Least deprived)	14,592 (21.6)	45,321 (19.3)	
2	13,891 (20.5)	45,917 (19.5)	
3	12,979 (19.2)	46,133 (19.6)	
4	12,559 (18.6)	46,018 (19.6)	
5 (Most deprived)	12,760 (18.9)	45,453 (19.3)	
Missing	868 (1.3)	6,109 (2.6)	
Body mass index category			<0.001
Underweight (<18.5 kg/m^2^)	177 (0.3)	961 (0.4)	
Healthy weight (18.5–24.9 kg/m^2^)	16,479 (24.4)	63,312 (26.9)	
Overweight (25–29.9 kg/m^2^)	14,930 (22.1)	51,523 (21.9)	
Obese (>30 kg/m^2^)	35,906 (53.1)	117,091 (49.8)	
Missing	157 (0.2)	2,064 (0.9)	
Charlson comorbidity score			<0.001
0	28,257 (41.8)	110,029 (46.8)	
1	16,367 (24.2)	50,767 (21.6)	
2	8,291 (12.3)	25,929 (11.0)	
3	6,857 (10.1)	20,377 (8.7)	
≥ 4	7,877 (11.6)	27,849 (11.9)	
Medical history (ICD-10 diagnosis)^*a*^			
Acute cerebrovascular disease	1,272 (1.9)	4,942 (2.1)	<0.001
Acute myocardial infarction	430 (0.6)	1,920 (0.8)	<0.001
Alcohol and substance-related disorders	9,670 (14.3)	38,386 (16.3)	<0.001
Any cancer or malignancy	25,267 (37.4)	75,374 (32.1)	<0.001
Aortic and peripheral arterial embolism or thrombosis	48 (0.1)	328 (0.1)	<0.001
Asthma	4,953 (7.3)	15,417 (6.6)	<0.001
Benign prostatic hyperplasia	11,087 (16.4)	35,040 (14.9)	<0.001
Cardiac dysrhythmias	8,542 (12.6)	35,096 (14.9)	<0.001
Chronic kidney disease	2,880 (4.3)	11,639 (5.0)	<0.001
Chronic obstructive pulmonary disease and bronchiectasis	7,531 (11.1)	27,124 (11.5)	0.003
Congestive heart failure	2,745 (4.1)	12,108 (5.2)	<0.001
Coronary atherosclerosis and other heart disease	9,160 (13.5)	31,399 (13.4)	0.235
Delirium, dementia, and other cognitive disorders	2,365 (3.5)	10,008 (4.3)	<0.001
Diabetes with or without chronic complications	27,069 (40.0)	84,347 (35.9)	<0.001
Epilepsy	867 (1.3)	3,612 (1.5)	<0.001
HIV infection	775 (1.1)	1,793 (0.8)	<0.001
Hypertension	38,232 (56.5)	119,796 (51.0)	<0.001
Influenza	541 (0.8)	1,340 (0.6)	<0.001
Mild liver diseases	4,403 (6.5)	14,502 (6.2)	0.001
Mental health conditions	28,588 (42.3)	106,727 (45.4)	<0.001
Osteoarthritis	14,097 (20.8)	42,326 (18.0)	<0.001
Peripheral and visceral atherosclerosis	2,472 (3.7)	9,168 (3.9)	0.003
Pneumonia	1,522 (2.2)	5,902 (2.5)	<0.001
Pulmonary heart disease	1,254 (1.9)	6,866 (2.9)	<0.001
Rheumatoid arthritis	1,312 (1.9)	4,012 (1.7)	<0.001
Septicemia	593 (0.9)	2,254 (1.0)	0.049
Thyroid disorder	7,565 (11.2)	24,075 (10.2)	<0.001
Tuberculosis	74 (0.1)	217 (0.1)	0.208
Immunocompromised^*b*^	13,347 (19.7)	43,751 (18.6)	<0.001
Smoking status			<0.001
Current or former	31,841 (47.1)	105,745 (45.0)	
Never	24,771 (36.6)	82,691 (35.2)	
Unknown	11,038 (16.3)	46,515 (19.8)	
VA frailty index (VA-FI)^*c*^			<0.001
Non-frail (VA-FI *<*0.1)	31,975 (47.3)	117,962 (50.2)	
Pre-frail (VA-FI >0.1–0.2)	20,638 (30.5)	64,150 (27.3)	
Mildly frail (VA-FI >0.2–0.3)	9,613 (14.2)	31,230 (13.3)	
Moderately frail (VA-FI >0.3–0.4)	3,613 (5.3)	13,484 (5.7)	
Severely frail (VA-FI >0.5)	1,810 (2.7)	8,125 (3.5)	
Outpatient visit 1 day prior to or on the index date	66,719 (98.6)	220,010 (93.6)	<0.001
Healthcare exposures, 1 year prior			
Hospital admission	6,337 (9.4)	22,998 (9.8)	0.001
Nursing home admission	365 (0.5)	1,737 (0.7)	<0.001
Intensive care unit admission	985 (1.5)	4,135 (1.8)	<0.001
Emergency department visit	27,131 (40.1)	84,439 (35.9)	<0.001
Urgent care visit	4,381 (6.5)	11,916 (5.1)	<0.001
Emergency department or urgent care visits	30,334 (44.8)	93,031 (39.6)	<0.001
Primary care visit	65,473 (96.8)	221,728 (94.4)	<0.001
Prior medication exposures^*d*^			
Antibiotics	8,463 (12.5)	27,493 (11.7)	<0.001
Alpha 1 antagonist	9,776 (14.5)	32,137 (13.7)	<0.001
Antiarrhythmic	114 (0.2)	1,587 (0.7)	<0.001
Anticholinergic	245 (0.4)	902 (0.4)	0.417
Anticoagulant/antiplatelet	2,491 (3.7)	18,988 (8.1)	<0.001
Antifungal	<5 (<0.0)	9 (0.0)	0.739
Anti-inflammatory	847 (1.3)	2,814 (1.2)	0.254
HMG-CoA reductase inhibitor	30,248 (44.7)	90,920 (38.7)	<0.001
Opioid analgesic	3,843 (5.7)	13,282 (5.7)	0.783
Phosphodiesterase 5 inhibitors	10,711 (15.8)	32,670 (13.9)	<0.001
Sedative	399 (0.6)	1,543 (0.7)	0.055
Sedative/hypnotic/psychiatric	8,262 (12.2)	35,880 (15.3)	<0.001
COVID-19 treatment guideline interaction categories (15, 16)			
Use an alternate drug	10,724 (15.9)	32,803 (14.0)	<0.001
Hold drug	32,782 (48.5)	102,511 (43.6)	<0.001
Dose adjust drug	35,096 (51.9)	120,655 (51.3)	0.016
Monitor drug	12,717 (18.8)	46,969 (20.0)	<0.001
COVID vaccination status^*e*^			<0.001
Up-to-date	25,170 (37.2)	72,568 (30.9)	
Vaccinated, but not up-to-date	34,115 (50.4)	114,876 (48.9)	
Unvaccinated	8,364 (12.4)	47,507 (20.2)	
Time since last COVID-19 vaccine (quintile)			<0.001
1 (least time elapsed since the last COVID-19 vaccine)	12,447 (18.4)	34,733 (14.8)	
2	11,562 (17.1)	39,000 (16.6)	
3	12,170 (18.0)	37,811 (16.1)	
4	10,977 (16.2)	39,092 (16.6)	
5 (most time elapsed since last COVID-19 vaccine)	12,129 (17.9)	36,808 (15.7)	
No vaccine	8,364 (12.4)	47,507 (20.2)	
Influenza vaccine in the past year	41,368 (61.2)	125,381 (53.4)	<0.001
Pneumococcal vaccine in the last 5 years	26,711 (39.5)	80,471 (34.3)	<0.001
Prior COVID-19 infection	6,708 (9.9)	28,132 (12.0)	<0.001
Pre delta	2,772 (4.1)	10,952 (4.7)	<0.001
Delta	1,346 (2.0)	5,572 (2.4)	<0.001
Omicron	2,816 (4.2)	12,741 (5.4)	<0.001
History of infections (ICD-10 diagnosis, 30 days prior to index date)			
Acute respiratory infections	2,215 (3.3)	9,713 (4.1)	<0.001
Urinary tract infection	345 (0.5)	1,370 (0.6)	0.026
Pneumonia	143 (0.2)	642 (0.3)	0.005
Skin and soft tissue	332 (0.5)	1,115 (0.5)	0.590
Current infection (ICD-10 diagnosis, time varying within 7 days of index date)			
Urinary tract infection	308 (0.5)	1,608 (0.7)	<0.001
Pneumonia	268 (0.4)	2,129 (0.9)	<0.001
Skin and soft tissue	127 (0.2)	807 (0.3)	<0.001

^*a*^ Medical history included underlying conditions and diagnoses in the year prior to the index date, identified using ICD-10 codes.

^*b*^ Immunocompromised status was based on immunocompromised conditions in the year prior to the index date and immunosuppressive medications in the 90 days prior to the index date based on a slightly modified and previously described algorithm (20). Unlike the previously described algorithm, we used diagnosis codes to identify solid organ or hematopoietic stem cell transplantation and HIV/AIDs vs patient registries. Consistent with the previously described algorithm, we required one inpatient or two outpatient diagnosis code for an immunocompromising condition (leukemia, lymphoma, congenital immunodeficiencies, asplenia/hyposplenia, HIV/AIDS, and organ transplant) in the year prior and any immunosuppressive medication (alkylating agents, antibiotics, antimetabolites, antimitotics, monoclonal antibodies, other, immune-modulating agents, TNF Alpha antagonist, and steroids) with an outpatient days supply or inpatient administration in the 90 days prior to the index date (20).

^*c*^ VA frailty index was categorized as non-frail (VA-FI ≤ 0.1), prefrail (>0.1–0.2), mildly frail (>0.2–0.3), moderately frail (>0.3–0.4), and severely frail (>0.4).

^*d*^ We assessed drugs with clinically significant potential drug-drug interactions with NMV/r (with recommendations to adjust concomitant medication and monitor, or to temporarily withhold concomitant medication, if clinically appropriate) (15, 16). Those on contraindicated medications were excluded: Alpha 1 antagonists (tamsulosin), antiarrhythmics (digoxin and ranolazine), anticholinergics (solifenacin and tolterodine), anticoagulant/antiplatelets (apixaban, rivaroxaban, and ticagrelor), antifungals (ketoconazole), anti-inflammatory drugs (colchicine, dexamethasone doses above 16 mg, and sulfasalazine), HMG-CoA reductase inhibitors (atorvastatin, simvastatin, rosuvastatin, and lovastatin), opioid analgesics (hydrocodone and hydrocodone containing combinations, oxycodone and oxycodone containing combinations), phosphodiesterase 5 inhibitors (sildenafil and tadalafil), sedative/hypnotic/psychiatrics (trazodone, alprazolam, clonazepam, buspirone, quetiapine, aripiprazole, and guanfacine).

^*e*^ Up-to-date COVID-19 vaccination status: at least one dose of XBB vaccine if the index date occurred between 25 September 2023 and 31 March 2024, at least one dose of a BA.4/5 bivalent vaccine if the index date occurred between 1 September 2022 and 24 September 2023, or at least three doses of wild-type COVID-19 vaccine if the index date occurred between 1 April 2022 and 31 August 2022.

^*f*^ ICD-10 = International Classification of Diseases, Tenth Revision. Data are *n* (%). *χ*^2^ or Fisher’s Exact tests were used to compare differences in proportions between the groups. For continuous variables, comparisons were performed using the Wilcoxon Rank Sum test or a Student’s *t*-test, depending on the distribution of the data for the given variable.

**Fig 2:**
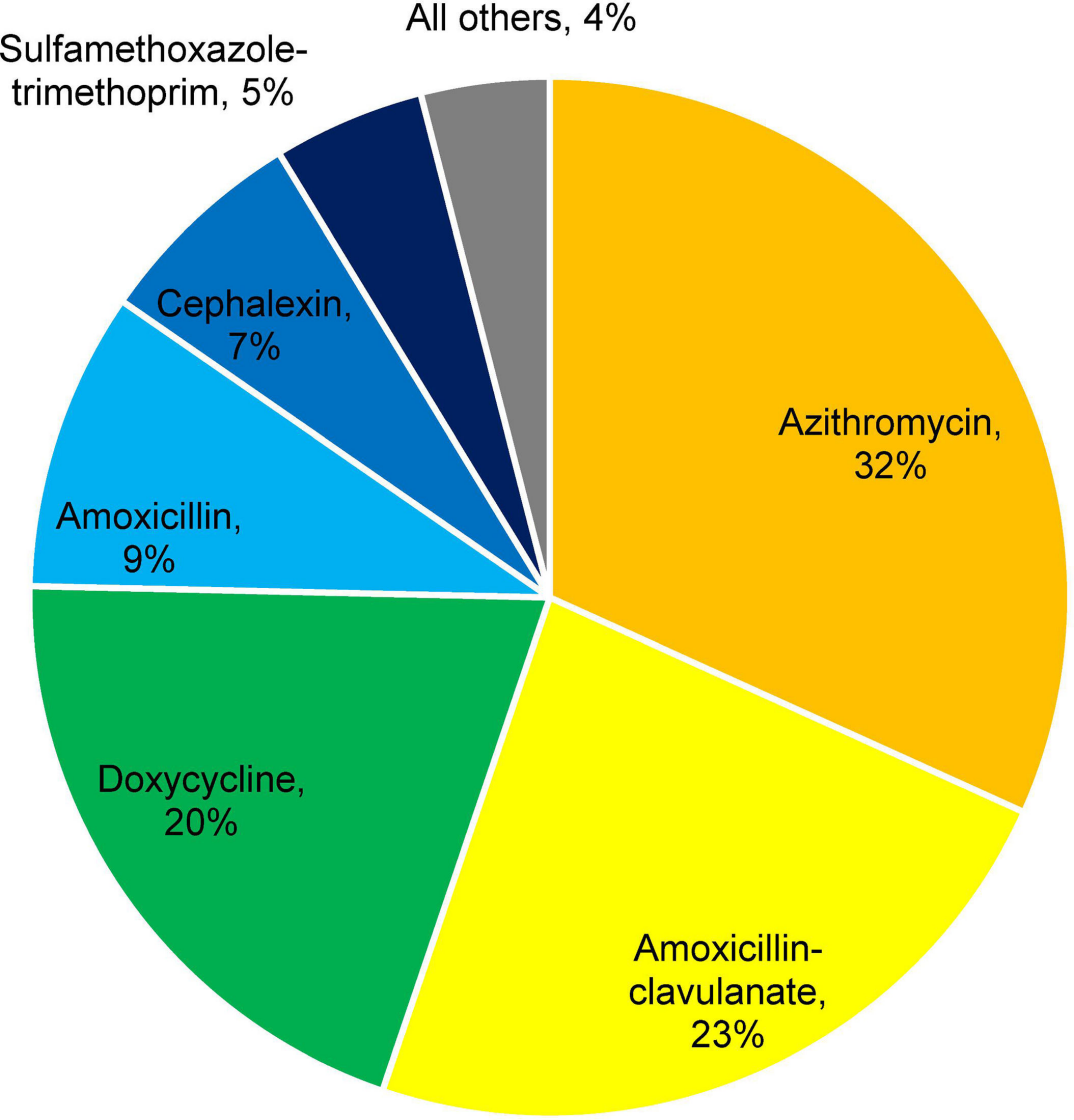
Initial outpatient antibiotic prescribed in the 30 days after COVID-19.

Overall, 26,434 of 302,600 (8.7%) received an antibiotic in the outpatient setting in the first 30 days following either a positive SARS-CoV-2 test or COVID-19 diagnosis. The most prescribed antibiotics were azithromycin, amoxicillin-clavulanate, doxycycline, and amoxicillin (Fig. 2). Among those who received an outpatient antibiotic, the median time between the index date and antibiotic treatment initiation was longer in the NMV/r-treated group compared to those who did not receive NMV/r (5 vs 0 days, respectively; *P* < 0.001). Patients who received outpatient antibiotics were generally similar to those who did not receive outpatient antibiotics in terms of age, sex, race, and ethnicity. Patients who received outpatient antibiotics (vs those who did not) were more likely to have been prescribed antibiotics in the prior 30 days (22.7% vs 10.8%, respectively, *P* < 0.001), be frail (59.7% vs 49.6%, *P* < 0.001), have a Charlson comorbidity score ≥1 (61.6% vs 53.6%, *P* < 0.001), have chronic obstructive pulmonary disease (17.9% vs 10.8%, *P* < 0.001), have visited the emergency department in the prior year (49.0% vs 35.7%, *P* < 0.001), and be immunocompromised (35.4% vs 17.3%, *P* < 0.001; Table S3).

Outpatient antibiotic receipt differed by NMV/r treatment status, with 7.2% of NMV/r-treated patients (4,901/67,649) prescribed outpatient antibiotics, compared with 9.2% of NMV/r-untreated patients (21,533/234,951). This corresponded to an adjusted hazard ratio (HR) of 0.65 (95% CI: 0.63‒0.68; Table 2).

**TABLE 2:** Cox proportional hazards regression evaluating the association between receipt of NMV/r and outpatient antibiotic prescriptions in the 30 days following COVID-19^*a*^^,^

Analysis and treatment group	Proportion who received outpatient antibiotics *n*/*N* (%)	Crude HR (95% CI)	Adj HR (95% CI)^*b*^
Primary analysis			
Received NMV/r	4,901/67,649 (7.2)	0.70 (0.68–0.72)	0.65 (0.63–0.68)
Did not receive NMV/r	21,533/234,951 (9.2)	Ref	Ref
Sensitivity analysis excluding patients who received an antibiotic prescription on the index date^*a*^			
Received NMV/r	2,946/65,694 (4.5)	0.97 (0.93–1.01)	0.91 (0.87–0.95)
Did not receive NMV/r	9,980/223,398 (4.5)	Ref	Ref

^*a*^ The index date was the date of positive SARS-CoV-2 test or COVID-19 diagnosis, whichever occurred first.

^*b*^ Cox proportional hazards models were adjusted for the following covariates: week of SARS-CoV-2 infection or COVID-19 diagnosis, whether an outpatient visit occurred at the time of testing positive or COVID-19 diagnosis (yes/no) (8), age group (18‒49, 50‒64, 65‒74, 75‒84, and ≥85 years), sex (male or female), race (Black or African American, White, and other), ethnicity (Hispanic or Latino and not Hispanic or Latino), region (Midwest, Northeast, West, and South), socioeconomic indicators (measured by the area deprivation index [ADI] grouped into quintiles from least to most deprived) (18), body mass index category (underweight [<18.5], normal weight [18.5‒24.9], overweight [25.0‒29.9], obese [≥30.0], or missing) (19), Charlson comorbidity index (0, 1, 2, 3, and ≥4), history of medical conditions (yes/no; modeled individually), including cancer, asthma, chronic obstructive pulmonary disease, hypertension, congestive heart failure, atherosclerosis, or other heart disease, cognitive disorders, including dementia, diabetes, HIV/AIDS, or liver disease, history of immunocompromising conditions, or use of immunosuppressive treatment (yes/no) (20), smoking status (current or former, never, and unknown), prior healthcare interactions (primary care visit in the previous year [yes/no], hospitalization in the previous year [yes/no], and emergency department or urgent care visit in the previous year [yes/no]), use of medications with the potential for drug-drug interactions with NMV/r in the 90 days prior to index (use an alternate COVID-19 therapy, hold drug, dose adjust drug, monitor drug, or not on concomitant medication with DDI potential) (16, 21), antibiotic use in the 30 days prior to index (yes/no), COVID-19 vaccination status (up-to-date [defined as receiving at least one dose of XBB vaccine if the index date occurred between 25 September 2023 and 31 March 2024, at least one dose of a BA.4/5 bivalent vaccine if the index date occurred between 1 September 2022 and 24 September 2023, or at least three doses of wild-type COVID-19 vaccine if the index date occurred between 1 April 2022 and 31 August 2022], previously vaccinated against COVID-19 but not up-to-date, and never received COVID-19 vaccine), receipt of influenza vaccine in the previous year (yes/no), receipt of pneumococcal vaccine in the previous 5 years (yes/no), prior documented SARS-CoV-2 infection (yes/no), current (index date to 7 days post index) urinary tract infection (time-varying), history of urinary tract infection in the 30 days prior to index (yes/no), and history of acute respiratory infection in the 30 days prior to index (yes/no).

In a sensitivity analysis that excluded patients who received an antibiotic prescription on the index date (i.e., likely empiric therapy), the relationship between NMV/r receipt and outpatient antibiotic prescribing persisted but was attenuated (adjusted HR: 0.91, 95% CI: 0.87‒0.95; Table 2). The findings utilizing propensity score matching (adjusted HR: 0.67, 95% CI: 0.64–0.70; Tables S4 and S5) and restricting the analyses to those with positive SARS-CoV-2 lab tests (adjusted HR: 0.63, 95% CI: 0.60‒0.65; Table S5) were similar to the primary analysis.

## DISCUSSION

Our study found that NMV/r-eligible patients with COVID-19 who received NMV/r were 35% (95% CI: 32%‒37%) less likely to be prescribed an antibiotic in the outpatient setting in the 30 days following a positive SARS-CoV-2 test or COVID-19 diagnosis compared to those who did not receive NMV/r. This difference was most pronounced when including antibiotic treatment dispensed at the time of COVID-19 diagnosis, suggesting that our results were possibly driven by a diminished perceived need for empiric antibiotic therapy when NMV/r is prescribed. This is consistent with our finding that antibiotic prescribing tended to occur on the same day as COVID-19 diagnosis (i.e., likely empiric therapy) among those who were prescribed an antibiotic and did not receive NMV/r but tended to occur later in the course of illness (i.e., antibiotic receipt a median of 5 days after COVID-19 diagnosis) among the smaller number of patients who received both antibiotics and NMV/r.

The absolute risk reduction of outpatient antibiotic prescribing for NMV/r-treated patients was 1.9%, meaning that, if the observed association were causal, for every 53 patients treated with NMV/r, one COVID-19-related antibiotic prescription (that was likely unnecessary [1, 22]) was prevented. These findings highlight the potential role of NMV/r in not only treating COVID-19 but also in supporting antibiotic stewardship and potentially combating antibiotic resistance in the aftermath of the COVID-19 pandemic. These results have two major implications for clinical practice. First, clinicians should prioritize appropriate treatment of COVID-19 with antivirals, which remain underutilized (23–28). Second, clinicians should carefully review signs and symptoms of infection in patients with COVID-19 and use antibiotics only in patients with high suspicion of bacterial co-infection. Antibiotic stewardship efforts should emphasize that bacterial co-infection in patients with COVID-19 is uncommon, making empiric antibiotic therapy often unnecessary (1–6).

Our findings complement the growing body of research on antibiotic use in patients with COVID-19. During the pandemic, antibiotics were often unnecessarily prescribed for COVID-19 cases (2, 3), likely driven by the initial lack of effective COVID-19 treatment options and fears about the potential for secondary bacterial infections. Numerous publications have shown increases in antibacterial prescribing rates during and after the COVID-19 pandemic, including significant increases in antibiotic prescriptions among patients with COVID-19 (1–3). Data indicate that antibiotic use in patients with COVID-19 likely ranges from 13% to 30% in outpatients (29, 30) and 62% to 72% among hospitalized patients (22, 31), despite the fact that secondary bacterial infection in patients with COVID-19 is relatively rare (<5%) (1, 2, 4–6, 22). A potential added benefit of NMV/r treatment may be in reducing the reliance on empiric antibiotic therapy in the outpatient management of COVID-19, which could have broad implications for reducing unnecessary antibiotic use and improving antibiotic stewardship.

Our study has several important limitations. We defined COVID-19 based on a positive SARS-CoV-2 laboratory test or COVID-19 diagnosis code, which can be subject to misclassification or omission. However, results were similar when only including patients with positive tests. In addition, testing, diagnosis, or treatment for COVID-19 (including prescriptions for antivirals or antibiotics) that occurred outside of the VA Healthcare system may not have been fully captured. To mitigate this issue, however, our study focused on active VA users who are more likely to rely on the VA Healthcare System for care. Furthermore, although we controlled for a wealth of sociodemographic and clinical characteristics between those who did and did not receive NMV/r, residual confounding due to unmeasured or unknown factors is possible, including the severity of symptoms at the time of diagnosis. Similarly, while we controlled for the region, regional differences in antibiotic prescribing practices, particularly the higher rates of antibiotic use observed in the South, may still contribute to the observed differences (32). Another limitation is that we only assessed outpatient antibiotic prescribing; therefore, our findings do not apply to inpatient antibiotic use, and future studies should explore this. Additionally, our data reflect antibiotic dispensing, and therefore, we cannot ascertain whether they were taken as prescribed. Finally, the generalizability of our findings also may be limited, as the US Veteran population predominantly comprises older males.

### Conclusions

In conclusion, our study suggests that the availability of effective SARS-CoV-2 antiviral treatment, specifically NMV/r, may influence outpatient antibiotic prescribing patterns. NMV/r-eligible patients with COVID-19 who received NMV/r were 35% less likely to be prescribed outpatient antibiotics compared to NMV/r-eligible patients who did not receive NMV/r. This difference was most pronounced when including antibiotic treatment dispensed at the time of COVID-19 diagnosis, suggesting that the observed difference may be influenced by a diminished perceived need for empiric antibiotic therapy when NMV/r is prescribed. Lower rates of antibiotic prescribing among patients treated with NMV/r may reflect a reduction in unnecessary outpatient antibiotic use. These findings highlight the potential added benefit of SARS-CoV-2 antiviral therapy in supporting antibiotic stewardship, though further investigation is needed. Finally, more educational efforts are needed for clinicians to emphasize that bacterial co-infection in patients with COVID-19 is uncommon, and empiric antibiotic therapy is likely unnecessary.

## Data Availability

The data supporting the findings of this study are not publicly available due to the inclusion of identifiable protected health information from the Veterans Health Administration. Privacy regulations prevent the open sharing of the individual-level data used in this study, and any data covered under these regulations cannot be shared. The Veterans Health Administration may approve the sharing of some study data after verifying de-identification, though this may not include all final study data. Each request is subject to approval by the ethics board, privacy office, and information systems and security office. For such requests, please contact the corresponding author.

## SUPPLEMENTAL MATERIAL

Supplemental tables spectrum.03209-24-s0001.docx Tables S1 to S5.

ASM does not own the copyrights to Supplemental Material that may be linked to, or accessed through, an article. The authors have granted ASM a non-exclusive, world-wide license to publish the Supplemental Material files. Please contact the corresponding author directly for reuse.
