# Supplementary material for: Association between nirmatrelvir/ritonavir treatment and antibiotic prescribing in the outpatient setting among patients with COVID-19
Source: Microbiol Spectr. 2025 Mar 5;13(4):e03209-24. doi: 10.1128/spectrum.03209-24 (PMC11960072; doi:10.1128/spectrum.03209-24)
Supplement: Supplemental tables — Tables S1 to S5. [file spectrum.03209-24-s0001.docx]

SUPPLEMENTAL MATERIAL

Supplemental Table 1: Drugs contraindicated with NMV/r that require the use of an alternative COVID-19 therapy

| Albuvirtide | Eplerenone | Pimozide |
| --- | --- | --- |
| Amiodarone | Ergometrine (Ergonovine) | Primidone |
| Apalutamide | Ergotamine | Propafenone |
| Avapritinib | Flecainide | Quinidine |
| Bepridil | Glecaprevir/pibrentasvir | Reboxetine |
| Bexarotene | Halofantrine | Rifampicin |
| Bosentan | Ibrutinib | Rifapentine |
| Carbamazepine | Infigratinib | Sonidegib |
| Cenobamate | Ivabradine | Sotorasib |
| Cisapride | Ivacaftor/Lumacaftor | St John's Wort |
| Clopidogrel | Ivosidenib | Tepotinib |
| Clozapine | Lorlatinib | Tolvaptan |
| Dabrafenib | Lurasidone | Toremifene |
| Dasatinib | Mefloquine | Tranylcypromine |
| Dextropropoxyphene | Methylergometrine (Methylergonovine) | Umbralisib |
| Dihydroergotamine | Midazolam (Oral) | Vardenafil |
| Disopyramide | Mitotane | Venetoclax |
| Dofetilide | Nilotinib | Voclosporin |
| Dronedarone | Pexidartinib |  |
| Enasidenib | Phenobarbital (Phenobarbitone) |  |
| Enzalutamide | Phenytoin |  |

Supplemental Table 2: Assessed outpatient antibiotics commonly used to treat respiratory tract infections

| Amoxicillin | Clindamycin |
| --- | --- |
| Amoxicillin-clavulanate | Dicloxacillin |
| Azithromycin | Doxycycline |
| Cefadroxil | Levofloxacin |
| Cefdinir | Linezolid |
| Cefpodoxime | Minocycline |
| Cefuroxime | Moxifloxacin |
| Cephalexin | Penicillin |
| Ciprofloxacin | Sulfamethoxazole-trimethoprim |
| Clarithromycin | Tetracycline |

Supplemental Table 3. Patient characteristics by receipt of outpatient antibiotic in 30 days following a positive SARS-CoV-2 test or COVID-19 diagnosis

|  | Received outpatient antibiotic (n=26,434) | Did not receive outpatient antibiotic (n=276,166) | P value |
| --- | --- | --- | --- |
| Variant time period |  |  | <0.001 |
| Pre-XBB Omicron | 15,091 (57.1) | 175,253 (63.5) |  |
| XBB | 8,230 (31.1) | 77,270 (28.0) |  |
| JN.1 | 3,113 (11.8) | 23,643 (8.6) |  |
| Age group |  |  | <0.001 |
| 18-49 years | 5,530 (20.9) | 63,818 (23.1) |  |
| 50-64 years | 7,742 (29.3) | 79,365 (28.7) |  |
| 65-74 years | 7,123 (26.9) | 69,341 (25.1) |  |
| 75-84 years | 4,918 (18.6) | 50,571 (18.3) |  |
| >85 years | 1,121 (4.2) | 13,071 (4.7) |  |
| Sex |  |  | <0.001 |
| Male | 22,004 (83.2) | 235,747 (85.4) |  |
| Female | 4,430 (16.8) | 40,419 (14.6) |  |
| Race |  |  | <0.001 |
| Black or African American | 5,978 (22.6) | 58,660 (21.2) |  |
| White | 17,647 (66.8) | 184,179 (66.7) |  |
| Other race | 2,809 (10.6) | 33,327 (12.1) |  |
| Ethnicity |  |  | <0.001 |
| Hispanic or Latino | 2,539 (9.6) | 28,399 (10.3) |  |
| Not Hispanic or Latino | 23,895 (90.4) | 247,767 (89.7) |  |
| Region |  |  | <0.001 |
| Midwest | 5,022 (19.0) | 55,100 (20.0) |  |
| Northeast | 3,414 (12.9) | 43,088 (15.6) |  |
| West | 4,822 (18.2) | 65,089 (23.6) |  |
| South | 13,176 (49.8) | 112,889 (40.9) |  |
| Area deprivation index (ADI^18^) Quintile |  |  | <0.001 |
| 1 (Least Deprived) | 4,336 (16.4) | 55,577 (20.1) |  |
| 2 | 4,893 (18.5) | 54,915 (19.9) |  |
| 3 | 5,244 (19.8) | 53,868 (19.5) |  |
| 4 | 5,444 (20.6) | 53,133 (19.2) |  |
| 5 (Most Deprived) | 6,067 (23.0) | 52,146 (18.9) |  |
| Missing | 450 (1.7) | 6,527 (2.4) |  |
| Body mass index category |  |  | <0.001 |
| Underweight (<18.5 kg/m^2^) | 127 (0.5) | 1,011 (0.4) |  |
| Healthy weight (18.5–24.9 kg/m^2^) | 6,816 (25.8) | 72,975 (26.4) |  |
| Overweight (25–29.9 kg/m^2^) | 5,673 (21.5) | 60,780 (22.0) |  |
| Obese (>30 kg/m^2^) | 13,759 (52.1) | 139,238 (50.4) |  |
| Missing | 59 (0.2) | 2,162 (0.8) |  |
| Charlson comorbidity score |  |  | <0.001 |
| 0 | 10,148 (38.4) | 128,138 (46.4) |  |
| 1 | 6,097 (23.1) | 61,037 (22.1) |  |
| 2 | 3,411 (12.9) | 30,809 (11.2) |  |
| 3 | 2,704 (10.2) | 24,530 (8.9) |  |
| ≥ 4 | 4,074 (15.4) | 31,652 (11.5) |  |
| Medical history (ICD-10 diagnosis)* |  |  |  |
| Acute cerebrovascular disease | 579 (2.2) | 5,635 (2.0) | 0.101 |
| Acute myocardial infarction | 247 (0.9) | 2,103 (0.8) | 0.002 |
| Alcohol and substance related disorders | 4,778 (18.1) | 43,278 (15.7) | <0.001 |
| Any cancer or malignancy | 9,861 (37.3) | 90,780 (32.9) | <0.001 |
| Aortic and peripheral arterial embolism or thrombosis | 47 (0.2) | 329 (0.1) | 0.010 |
| Asthma | 2,317 (8.8) | 18,053 (6.5) | <0.001 |
| Benign prostatic hyperplasia | 4,851 (18.4) | 41,276 (14.9) | <0.001 |
| Cardiac dysrhythmias | 4,508 (17.1) | 39,130 (14.2) | <0.001 |
| Chronic kidney disease | 1,504 (5.7) | 13,015 (4.7) | <0.001 |
| Chronic obstructive pulmonary disease and bronchiectasis | 4,728 (17.9) | 29,927 (10.8) | <0.001 |
| Congestive heart failure | 1,687 (6.4) | 13,166 (4.8) | <0.001 |
| Coronary atherosclerosis and other heart disease | 4,156 (15.7) | 36,403 (13.2) | <0.001 |
| Delirium, dementia, and other cognitive disorders | 1,136 (4.3) | 11,237 (4.1) | 0.073 |
| Diabetes with or without chronic complications | 10,620 (40.2) | 100,796 (36.5) | <0.001 |
| Epilepsy | 429 (1.6) | 4,050 (1.5) | 0.044 |
| Human immunodeficiency virus (HIV) infection | 269 (1.0) | 2,299 (0.8) | 0.002 |
| Hypertension | 14,816 (56.0) | 143,212 (51.9) | <0.001 |
| Influenza | 260 (1.0) | 1,621 (0.6) | <0.001 |
| Mild liver diseases | 1,945 (7.4) | 16,960 (6.1) | <0.001 |
| Mental health conditions | 12,855 (48.6) | 122,460 (44.3) | <0.001 |
| Osteoarthritis | 5,828 (22.0) | 50,595 (18.3) | <0.001 |
| Peripheral and visceral atherosclerosis | 1,357 (5.1) | 10,283 (3.7) | <0.001 |
| Pneumonia | 1,118 (4.2) | 6,306 (2.3) | <0.001 |
| Pulmonary heart disease | 896 (3.4) | 7,224 (2.6) | <0.001 |
| Rheumatoid arthritis | 585 (2.2) | 4,739 (1.7) | <0.001 |
| Septicemia | 446 (1.7) | 2,401 (0.9) | <0.001 |
| Thyroid disorder | 3,142 (11.9) | 28,498 (10.3) | <0.001 |
| Tuberculosis | 36 (0.1) | 255 (0.1) | 0.028 |
| Immunocompromised** | 9,355 (35.4) | 47,743 (17.3) | <0.001 |
| Smoking |  |  | <0.001 |
| Current or former | 12,616 (47.7) | 124,970 (45.3) |  |
| Never | 9,335 (35.3) | 98,126 (35.5) |  |
| Unknown | 4,483 (17.0) | 53,070 (19.2) |  |
| VA Frailty index (VA-FI)*** |  |  | <0.001 |
| Non-frail (VA-FI *<*0.1) | 10,641 (40.3) | 139,296 (50.4) |  |
| Pre-frail (VA-FI >0.1–0.2) | 7,791 (29.5) | 76,997 (27.9) |  |
| Mildly frail (VA-FI >0.2–0.3) | 4,504 (17.0) | 36,339 (13.2) |  |
| Moderately frail (VA-FI >0.3–0.4) | 2,166 (8.2) | 14,931 (5.4) |  |
| Severely frail (VA-FI >0.5) | 1,332 (5.0) | 8,603 (3.1) |  |
| Outpatient visit one day prior or on index date | 25,794 (97.6) | 260,935 (94.5) | <0.001 |
| Healthcare exposures, 1 year prior |  |  |  |
| Hospital admission | 3,554 (13.4) | 25,781 (9.3) | <0.001 |
| Nursing home admission | 219 (0.8) | 1,883 (0.7) | 0.006 |
| Intensive care unit admission | 597 (2.3) | 4,523 (1.6) | <0.001 |
| Emergency department visit | 12,946 (49.0) | 98,624 (35.7) | <0.001 |
| Urgent care visit | 1,913 (7.2) | 14,384 (5.2) | <0.001 |
| Emergency department or urgent care visits | 14,339 (54.2) | 109,026 (39.5) | <0.001 |
| Primary care visit | 25,558 (96.7) | 261,643 (94.7) | <0.001 |
| Prior medication exposures**** |  |  |  |
| Antibiotics | 6,012 (22.7) | 29,944 (10.8) | <0.001 |
| Alpha 1 antagonist | 4,522 (17.1) | 37,391 (13.5) | <0.001 |
| Antiarrhythmic | 185 (0.7) | 1,516 (0.5) | 0.002 |
| Anticholinergic | 150 (0.6) | 997 (0.4) | <0.001 |
| Anticoagulant/antiplatelet | 2,217 (8.4) | 19,262 (7.0) | <0.001 |
| Antifungal | <5 (<0.0) | 11 (0.0) | 0.305 |
| Anti-inflammatory | 347 (1.3) | 3,314 (1.2) | 0.109 |
| HMG-CoA reductase inhibitor | 11,282 (42.7) | 109,886 (39.8) | <0.001 |
| Opioid analgesic | 2,256 (8.5) | 14,869 (5.4) | <0.001 |
| Phosphodiesterase 5 inhibitors | 4,093 (15.5) | 39,288 (14.2) | <0.001 |
| Sedative | 227 (0.9) | 1,715 (0.6) | <0.001 |
| Sedative/hypnotic/psychiatric | 4,646 (17.6) | 39,496 (14.3) | <0.001 |
| COVID-19 Treatment Guideline Interaction Categories^15,16^ |  |  |  |
| Use alternate drug | 4,111 (15.6) | 39,416 (14.3) | <0.001 |
| Hold drug | 12,982 (49.1) | 122,311 (44.3) | <0.001 |
| Dose adjust drug | 15,604 (59.0) | 140,147 (50.7) | <0.001 |
| Monitor drug | 6,887 (26.1) | 52,799 (19.1) | <0.001 |
| COVID vaccination status***** |  |  | <0.001 |
| Up to date with vaccine | 7,747 (29.3) | 89,991 (32.6) |  |
| Vaccinated, but not up to date | 13,427 (50.8) | 135,564 (49.1) |  |
| Unvaccinated | 5,260 (19.9) | 50,611 (18.3) |  |
| Time since last COVID-19 vaccine (quintile) |  |  | <0.001 |
| 1 (Least time elapsed since last COVID-19 vaccine) | 3,867 (14.6) | 43,313 (15.7) |  |
| 2 | 3,933 (14.9) | 46,629 (16.9) |  |
| 3 | 4,079 (15.4) | 45,902 (16.6) |  |
| 4 | 4,450 (16.8) | 45,619 (16.5) |  |
| 5 (Most time elapsed since last COVID-19 vaccine) | 4,845 (18.3) | 44,092 (16.0) |  |
| No vaccine | 5,260 (19.9) | 50,611 (18.3) |  |
| Influenza vaccine in past year | 14,317 (54.2) | 152,432 (55.2) | 0.001 |
| Pneumococcal vaccine in last 5 years | 9,991 (37.8) | 97,191 (35.2) | <0.001 |
| Prior COVID-19 infection | 3,657 (13.8) | 31,183 (11.3) | <0.001 |
| Pre delta | 1,481 (5.6) | 12,243 (4.4) | <0.001 |
| Delta | 747 (2.8) | 6,171 (2.2) | <0.001 |
| Omicron | 1,588 (6.0) | 13,969 (5.1) | <0.001 |
| History of infection ICD-10 diagnosis (30 days prior to index date) |  |  |  |
| Acute respiratory infections | 1,544 (5.8) | 10,384 (3.8) | <0.001 |
| Urinary tract infection | 335 (1.3) | 1,380 (0.5) | <0.001 |
| Pneumonia | 120 (0.5) | 665 (0.2) | <0.001 |
| Skin and soft tissue | 256 (1.0) | 1,191 (0.4) | <0.001 |
| Current infection ICD-10 diagnosis (time-varying within 7 days of index date) |  |  |  |
| Urinary tract infection | 844 (3.2) | 1,072 (0.4) | <0.001 |
| Pneumonia | 1,022 (3.9) | 1,375 (0.5) | <0.001 |
| Skin and soft tissue | 476 (1.8) | 458 (0.2) | <0.001 |

ICD-10 = International Classification of Diseases, Tenth Revision.

Data are n (%). Chi-squared or Fisher’s Exact tests were used to compare differences in proportions between the groups. For continuous variables, comparisons were performed using the Wilcoxon Rank Sum test or a Student’s t-test, depending on the distribution of the data for the given variable.

*Medical history included underlying conditions and diagnoses in the year prior to the index date, identified using ICD-10 codes.

**Immunocompromised status was based on immunocompromised conditions in the year prior to the index date and immunosuppressive medications in the 90 days prior to the index date based on a slightly modified and previously described algorithm.^20^ Unlike the previously described algorithm, we used diagnosis codes to identify solid organ or hematopoietic stem cell transplantation and HIV/AIDs versus patient registries. Consistent with the previously described algorithm, we required one inpatient or two outpatient diagnosis code for an immunocompromising condition (leukemia, lymphoma, congenital immunodeficiencies, asplenia/hyposplenia, HIV/AIDS, and organ transplant) in the year prior and any immunosuppressive medication (alkylating agents, antibiotics, antimetbolites, antimitotics, monoclonal antibodies, other, immune-modulating agents, TNF Alpha antagonist, and steroids) with an outpatient days supply or inpatient administration in the 90 days prior to the index date.^20^

***VA Frailty index was categorized as non-frail (VA-FI ≤ 0.1), prefrail (>0.1–0.2), mildly frail (>0.2–0.3), moderately frail (>0.3–0.4), and severely frail (>0.4).

****Prior medication exposures included drugs with any days supply during the 90 days prior to the index date.

We assessed drugs with clinically significant potential drug-drug interactions with NMV/r (with recommendations to adjust concomitant medication and monitor, or to temporarily withhold concomitant medication, if clinically appropriate).^15,16^ Those on contraindicated medications were excluded.

Alpha 1 antagonists included tamsulosin.

Antiarrhythmics included digoxin and ranolazine.

Anticholinergics included solifenacin, and tolterodine.

Anticoagulant/antiplatelets included apixaban, rivaroxaban, and ticagrelor.

Antifungals included ketoconazole.

Anti-inflammatory drugs included colchicine, dexamethasone (doses above 16 mg), and sulfasalazine.

HMG-CoA reductase inhibitors included atorvastatin, simvastatin, rosuvastatin, and lovastatin.

Opioid analgesics included hydrocodone (and hydrocodone containing combinations) and oxycodone (and oxycodone containing combinations).

Phosphodiesterase 5 inhibitors sildenafil and tadalafil.

Sedative/hypnotic/psychiatrics included trazodone, alprazolam, clonazepam, buspirone, quetiapine, aripiprazole, and guanfacine.

*****Up-to-date COVID-19 vaccination status: at least one dose of XBB vaccine if the index date occurred between September 25, 2023 and March 31, 2024, at least one dose of a BA.4/5 bivalent vaccine if the index date occurred between September 1, 2022 and September 24, 2023, or at least three doses of wild-type COVID-19 vaccine if the index date occurred between April 1, 2022 and August 31, 2022.

Supplemental Table 4. Patient characteristics by receipt of nirmatrelvir/ritonavir (NMV/r) in propensity score matched cohort

|  | Received NMV/r (n=67,153) | Did not receive NMV/r (n=67,153) | Standardized difference |
| --- | --- | --- | --- |
| Received outpatient antibiotic in 30 days following a positive SARS-CoV-2 test or COVID-19 diagnosis |  |  | -0.08 |
| Yes | 4,863 (7.2) | 6,429 (9.6) |  |
| No | 62,290 (92.8) | 60,724 (90.4) |  |
| Variant time period |  |  | 0.03 |
| Pre-XBB Omicron | 36,614 (54.5) | 36,809 (54.8) |  |
| XBB | 23,300 (34.7) | 22,011 (32.8) |  |
| JN.1 | 7,239 (10.8) | 8,333 (12.4) |  |
| Age group |  |  | 0.05 |
| 18-49 years | 11,569 (17.2) | 11,671 (17.4) |  |
| 50-64 years | 19,879 (29.6) | 19,868 (29.6) |  |
| 65-74 years | 19,147 (28.5) | 19,036 (28.3) |  |
| 75-84 years | 13,571 (20.2) | 13,609 (20.3) |  |
| >85 years | 2,987 (4.4) | 2,969 (4.4) |  |
| Sex |  |  | 0.00 |
| Male | 57,505 (85.6) | 57,520 (85.7) |  |
| Female | 9,648 (14.4) | 9,633 (14.3) |  |
| Race |  |  | 0.00 |
| Black or African American | 14,062 (20.9) | 14,091 (21.0) |  |
| White | 45,546 (67.8) | 45,493 (67.7) |  |
| Other race | 7,545 (11.2) | 7,569 (11.3) |  |
| Ethnicity |  |  | 0.00 |
| Hispanic or Latino | 5,995 (8.9) | 6,067 (9.0) |  |
| Not Hispanic or Latino | 61,158 (91.1) | 61,086 (91.0) |  |
| Region |  |  | 0.00 |
| Midwest | 15,422 (23.0) | 15,258 (22.7) |  |
| Northeast | 10,687 (15.9) | 10,778 (16.0) |  |
| West | 16,280 (24.2) | 16,253 (24.2) |  |
| South | 24,764 (36.9) | 24,864 (37.0) |  |
| Area deprivation index (ADI^18^) Quintile |  |  | 0.08 |
| 1 (Least Deprived) | 14,415 (21.5) | 14,340 (21.4) |  |
| 2 | 13,762 (20.5) | 13,835 (20.6) |  |
| 3 | 12,904 (19.2) | 12,847 (19.1) |  |
| 4 | 12,505 (18.6) | 12,533 (18.7) |  |
| 5 (Most Deprived) | 12,699 (18.9) | 12,709 (18.9) |  |
| Missing | 868 (1.3) | 889 (1.3) |  |
| Body mass index category |  |  | 0.00 |
| Underweight (<18.5 kg/m^2^) | 177 (0.3) | 163 (0.2) |  |
| Healthy weight (18.5–24.9 kg/m^2^) | 16,414 (24.4) | 16,419 (24.5) |  |
| Overweight (25–29.9 kg/m^2^) | 14,805 (22.0) | 14,852 (22.1) |  |
| Obese (>30 kg/m^2^) | 35,600 (53.0) | 35,576 (53.0) |  |
| Missing | 157 (0.2) | 143 (0.2) |  |
| Charlson comorbidity score |  |  | 0.00 |
| 0 | 28,157 (41.9) | 28,097 (41.8) |  |
| 1 | 16,203 (24.1) | 16,168 (24.1) |  |
| 2 | 8,214 (12.2) | 8,383 (12.5) |  |
| 3 | 6,781 (10.1) | 6,707 (10.0) |  |
| ≥ 4 | 7,798 (11.6) | 7,798 (11.6) |  |
| Medical history (ICD-10 diagnosis)* |  |  |  |
| Acute cerebrovascular disease | 1,266 (1.9) | 1,303 (1.9) | 0.00 |
| Acute myocardial infarction | 427 (0.6) | 447 (0.7) | 0.00 |
| Alcohol and substance related disorders | 9,637 (14.4) | 9,649 (14.4) | 0.00 |
| Any cancer or malignancy | 24,964 (37.2) | 25,059 (37.3) | 0.00 |
| Aortic and peripheral arterial embolism or thrombosis | 48 (0.1) | 52 (0.1) | 0.00 |
| Asthma | 4,905 (7.3) | 4,911 (7.3) | 0.00 |
| Benign prostatic hyperplasia | 10,981 (16.4) | 10,850 (16.2) | 0.01 |
| Cardiac dysrhythmias | 8,505 (12.7) | 8,420 (12.5) | 0.00 |
| Chronic kidney disease | 2,874 (4.3) | 2,892 (4.3) | 0.00 |
| Chronic obstructive pulmonary disease and bronchiectasis | 7,487 (11.1) | 7,422 (11.1) | 0.00 |
| Congestive heart failure | 2,735 (4.1) | 2,777 (4.1) | 0.00 |
| Coronary atherosclerosis and other heart disease | 9,082 (13.5) | 9,031 (13.4) | 0.00 |
| Delirium, dementia, and other cognitive disorders | 2,356 (3.5) | 2,386 (3.6) | 0.00 |
| Diabetes with or without chronic complications | 26,823 (39.9) | 26,732 (39.8) | 0.00 |
| Epilepsy | 864 (1.3) | 883 (1.3) | 0.00 |
| Human immunodeficiency virus (HIV) infection | 742 (1.1) | 767 (1.1) | 0.00 |
| Hypertension | 37,851 (56.4) | 37,918 (56.5) | 0.00 |
| Influenza | 525 (0.8) | 511 (0.8) | 0.00 |
| Mild liver diseases | 4,358 (6.5) | 4,469 (6.7) | -0.01 |
| Mental health conditions | 28,448 (42.4) | 28,584 (42.6) | 0.00 |
| Osteoarthritis | 13,923 (20.7) | 14,001 (20.8) | 0.00 |
| Peripheral and visceral atherosclerosis | 2,451 (3.6) | 2,432 (3.6) | 0.00 |
| Pneumonia | 1,510 (2.2) | 1,520 (2.3) | 0.00 |
| Pulmonary heart disease | 1,252 (1.9) | 1,218 (1.8) | 0.00 |
| Rheumatoid arthritis | 1,300 (1.9) | 1,315 (2.0) | 0.00 |
| Septicemia | 588 (0.9) | 598 (0.9) | 0.00 |
| Thyroid disorder | 7,500 (11.2) | 7,566 (11.3) | 0.00 |
| Tuberculosis | 73 (0.1) | 60 (0.1) | 0.01 |
| Immunocompromised** | 13,224 (19.7) | 13,334 (19.9) | 0.00 |
| Smoking status |  |  | 0.00 |
| Current or former | 31,590 (47.0) | 31,747 (47.3) |  |
| Never | 24,564 (36.6) | 24,595 (36.6) |  |
| Unknown | 10,999 (16.4) | 10,811 (16.1) |  |
| VA Frailty Index (VA-FI)*** |  |  | 0.06 |
| Non-frail (VA-FI *<*0.1) | 31,809 (47.4) | 31,715 (47.2) |  |
| Pre-frail (VA-FI >0.1–0.2) | 20,428 (30.4) | 20,551 (30.6) |  |
| Mildly frail (VA-FI >0.2–0.3) | 9,527 (14.2) | 9,462 (14.1) |  |
| Moderately frail (VA-FI >0.3–0.4) | 3,590 (5.3) | 3,645 (5.4) |  |
| Severely frail (VA-FI >0.5) | 1,799 (2.7) | 1,780 (2.7) |  |
| Outpatient visit one day prior or on index date | 66,223 (98.6) | 66,266 (98.7) | -0.01 |
| Healthcare exposures, 1 year prior |  |  |  |
| Hospital admission | 6,283 (9.4) | 6,228 (9.3) | 0.00 |
| Nursing home admission | 364 (0.5) | 365 (0.5) | 0.00 |
| Intensive care unit admission | 978 (1.5) | 985 (1.5) | 0.00 |
| Emergency department visit | 26,791 (39.9) | 26,807 (39.9) | 0.00 |
| Urgent care visit | 4,290 (6.4) | 4,172 (6.2) | 0.01 |
| Emergency department or urgent care visits | 29,923 (44.6) | 29,892 (44.5) | 0.00 |
| Primary care visit | 64,978 (96.8) | 65,010 (96.8) | 0.00 |
| Prior medication exposures**** |  |  |  |
| Antibiotics | 8,383 (12.5) | 8,031 (12) | 0.02 |
| Alpha 1 antagonist | 9,703 (14.4) | 9,673 (14.4) | 0.00 |
| Antiarrhythmic | 114 (0.2) | 115 (0.2) | 0.00 |
| Anticholinergic | 244 (0.4) | 241 (0.4) | 0.00 |
| Anticoagulant/antiplatelet | 2,491 (3.7) | 2,420 (3.6) | 0.01 |
| Antifungal | <5 (<0.0) | <5 (<0.0) | 0.00 |
| Anti-inflammatory | 841 (1.3) | 860 (1.3) | 0.00 |
| HMG-CoA reductase inhibitor | 29,923 (44.6) | 29,374 (43.7) | 0.02 |
| Opioid analgesic | 3,818 (5.7) | 3,825 (5.7) | 0.00 |
| Phosphodiesterase 5 inhibitors | 10,597 (15.8) | 10,579 (15.8) | 0.00 |
| Sedative | 398 (0.6) | 382 (0.6) | 0.00 |
| Sedative/hypnotic/psychiatric | 8,247 (12.3) | 8,481 (12.6) | -0.01 |
| COVID-19 Treatment Guideline Interaction Categories^15,16^ |  |  |  |
| Use alternate drug | 10,610 (15.8) | 10,610 (15.8) | 0.00 |
| Hold drug | 32,446 (48.3) | 32,480 (48.4) | 0.00 |
| Dose adjust drug | 34,812 (51.8) | 34,913 (52.0) | 0.00 |
| Monitor drug | 12,647 (18.8) | 12,832 (19.1) | -0.01 |
| COVID vaccination status***** |  |  | 0.00 |
| Up-to-date | 24,803 (36.9) | 24,807 (36.9) |  |
| Vaccinated, but not up-to-date | 33,987 (50.6) | 33,954 (50.6) |  |
| Unvaccinated | 8,363 (12.5) | 8,392 (12.5) |  |
| Time since last COVID-19 vaccine (quintile) |  |  | 0.00 |
| 1 (Least time elapsed since last COVID-19 vaccine) | 12,229 (18.2) | 12,183 (18.1) |  |
| 2 | 11,496 (17.1) | 11,440 (17) |  |
| 3 | 12,058 (18.0) | 11,998 (17.9) |  |
| 4 | 10,917 (16.3) | 10,948 (16.3) |  |
| 5 (Most time elapsed since last COVID-19 vaccine) | 12,090 (18.0) | 12,192 (18.2) |  |
| No vaccine | 8,363 (12.5) | 8,392 (12.5) |  |
| Influenza vaccine in past year | 40,917 (60.9) | 40,877 (60.9) | 0.00 |
| Pneumococcal vaccine in last 5 years | 26,442 (39.4) | 26,527 (39.5) | 0.00 |
| Prior COVID-19 infection | 6,693 (10.0) | 6,609 (9.8) | 0.00 |
| Pre delta | 2,765 (4.1) | 2,726 (4.1) | 0.00 |
| Delta | 1,346 (2.0) | 1,255 (1.9) | 0.01 |
| Omicron | 2,807 (4.2) | 2,888 (4.3) | -0.01 |
| History of infections (ICD-10 diagnosis, 30 days prior to index date) |  |  |  |
| Acute respiratory infections | 2,215 (3.3) | 2,230 (3.3) | 0.00 |
| Urinary tract infection | 343 (0.5) | 323 (0.5) | 0.00 |
| Pneumonia | 143 (0.2) | 154 (0.2) | 0.00 |
| Skin and soft tissue | 325 (0.5) | 297 (0.4) | 0.01 |
| Current infection (ICD-10 diagnosis, time-varying within 7 days of index date) |  |  |  |
| Urinary tract infection | 308 (0.5) | 328 (0.5) | 0.00 |
| Pneumonia | 268 (0.4) | 302 (0.4) | 0.00 |
| Skin and soft tissue | 126 (0.2) | 131 (0.2) | 0.00 |

ICD-10 = International Classification of Diseases, Tenth Revision.

Data are n (%).

*Medical history included underlying conditions and diagnoses in the year prior to the index date, identified using ICD-10 codes.

**Immunocompromised status was based on immunocompromised conditions in the year prior to the index date and immunosuppressive medications in the 90 days prior to the index date based on a slightly modified and previously described algorithm.^20^ Unlike the previously described algorithm, we used diagnosis codes to identify solid organ or hematopoietic stem cell transplantation and HIV/AIDs versus patient registries. Consistent with the previously described algorithm, we required one inpatient or two outpatient diagnosis code for an immunocompromising condition (leukemia, lymphoma, congenital immunodeficiencies, asplenia/hyposplenia, HIV/AIDS, and organ transplant) in the year prior and any immunosuppressive medication (alkylating agents, antibiotics, antimetbolites, antimitotics, monoclonal antibodies, other, immune-modulating agents, TNF Alpha antagonist, and steroids) with an outpatient days supply or inpatient administration in the 90 days prior to the index date.^20^

***VA Frailty index was categorized as non-frail (VA-FI ≤ 0.1), prefrail (>0.1–0.2), mildly frail (>0.2–0.3), moderately frail (>0.3–0.4), and severely frail (>0.4).

****Prior medication exposures included drugs with any days supply during the 90 days prior to the index date.

We assessed drugs with clinically significant potential drug-drug interactions with NMV/r (with recommendations to adjust concomitant medication and monitor, or to temporarily withhold concomitant medication, if clinically appropriate).^15,16^ Those on contraindicated medications were excluded.

Alpha 1 antagonists included tamsulosin.

Antiarrhythmics included digoxin and ranolazine.

Anticholinergics included solifenacin, and tolterodine.

Anticoagulant/antiplatelets included apixaban, rivaroxaban, and ticagrelor.

Antifungals included ketoconazole.

Anti-inflammatory drugs included colchicine, dexamethasone (doses above 16 mg), and sulfasalazine.

HMG-CoA reductase inhibitors included atorvastatin, simvastatin, rosuvastatin, and lovastatin.

Opioid analgesics included hydrocodone (and hydrocodone containing combinations) and oxycodone (and oxycodone containing combinations).

Phosphodiesterase 5 inhibitors sildenafil and tadalafil.

Sedative/hypnotic/psychiatrics included trazodone, alprazolam, clonazepam, buspirone, quetiapine, aripiprazole, and guanfacine.

*****Up-to-date COVID-19 vaccination status: at least one dose of XBB vaccine if the index date occurred between September 25, 2023 and March 31, 2024, at least one dose of a BA.4/5 bivalent vaccine if the index date occurred between September 1, 2022 and September 24, 2023, or at least three doses of wild-type COVID-19 vaccine if the index date occurred between April 1, 2022 and August 31, 2022.

Supplemental Table 5. Cox proportional hazards regression evaluating the association between receipt of NMV/r and outpatient antibiotic prescriptions in the 30 days following COVID-19*

| **Analysis and treatment group** | **Proportion who received outpatient antibiotics n / N (%)** | **Adj HR (95% CI)** |
| --- | --- | --- |
| Propensity score matched cohort |  |  |
| Received NMV/r | 4,863 / 67,153 (7.2) | 0.67 (0.64-0.70)** |
| Did not receive NMV/r | 6,429 / 67,153 (9.6) | ref |
| Including only patients with a positive SARS-CoV-2 lab test |  |  |
| Received NMV/r | 3,655 /38,325 (9.5) | 0.63 (0.60-0.65)*** |
| Did not receive NMV/r | 16,159 /138,755 (11.6) | ref |

CI = confidence interval; HR = hazard ratio; NMV/r = nirmatrelvir-ritonavir.

*The index date was the date of positive SARS-CoV-2 test or COVID-19 diagnosis, whichever occurred first.

**Matched on propensity score (PS) using nearest neighbor matching within a caliper of 0.0001. Variables included in the propensity score model: week of SARS-CoV-2 infection or COVID-19 diagnosis, whether an outpatient visit occurred at the time of testing positive or COVID-19 diagnosis (yes/no),^8^ age group (18‒49, 50‒64, 65‒74, 75‒84, and ≥85 years), sex (male or female), race (Black or African American, White, and other), ethnicity (Hispanic or Latino and not Hispanic or Latino), region (Midwest, Northeast, West, and South), socioeconomic indicators (measured by the area deprivation index [ADI] grouped into quintiles from least to most deprived),^18^ body mass index category (underweight [<18.5], normal weight [18.5‒24.9], overweight [25.0‒29.9], obese [≥30.0], or missing),^19^ Charlson comorbidity index (0, 1, 2, 3, and ≥4), history of medical conditions (yes/no; modeled individually) including acute myocardial infarction, alcohol and substance related disorders, any cancer or malignancy, aortic and peripheral arterial embolism or thrombosis, asthma, cardiac dysrhythmias, chronic kidney disease, chronic obstructive pulmonary disease and bronchiectasis, congestive heart failure/ no hypertensive, delirium, dementia, and amnestic and other cognitive disorders, epilepsy, human immunodeficiency virus (HIV) infection, hypertension, influenza, mental health conditions, multiple sclerosis, osteoarthritis, peripheral and visceral atherosclerosis, pulmonary heart disease, rheumatoid arthritis, septicemia, thyroid disorders, tuberculosis, history of immunocompromising conditions or use of immunosuppressive treatment (yes/no),^20^ smoking status (current or former, never, and unknown), prior healthcare interactions (primary care visit in the previous year [yes/no], hospitalization in the previous year [yes/no], emergency department or urgent care visit in the previous year [yes/no]), use of medications with the potential for drug-drug interactions with NMV/r in the 90 days prior to index (use an alternate COVID-19 therapy, hold drug, dose adjust drug, monitor drug, or not on concomitant medication with DDI potential),^16,21^ antibiotic use in the 30 days prior to index (yes/no), COVID-19 vaccination status (up-to-date [defined as receiving at least one dose of XBB vaccine if the index date occurred between September 25, 2023 and March 31, 2024, at least one dose of a BA.4/5 bivalent vaccine if the index date occurred between September 1, 2022 and September 24, 2023, or at least three doses of wild-type COVID-19 vaccine if the index date occurred between April 1, 2022 and August 31, 2022], previously vaccinated against COVID-19 but not up-to-date, and never received COVID-19 vaccine), receipt of influenza vaccine in the previous year (yes/no), receipt of pneumococcal vaccine in the previous 5 years (yes/no), prior documented SARS-CoV-2 infection (yes/no), current (index date to 7 days post index) urinary tract infection (time-varying), pneumonia (time-varying), skin and soft tissue infection (time-varying), history of pneumonia in the 30 days prior to index (yes/no), history of urinary tract infection in the 30 days prior to index (yes/no), and history of acute respiratory infection in the 30 days prior to index (yes/no), prior medication exposures in past 90 days alpha 1 antagonist, antiarrhythmic, anticoagulant/antiplatelet, anti-inflammatory, sedative/hypnotic/psychiatric, sedative and opioid analgesic and VA Frailty index was categorized as non-frail (VA-FI ≤ 0.1), prefrail (>0.1–0.2), mildly frail (>0.2–0.3), 420 moderately frail (>0.3–0.4), and severely frail (>0.4).

***Cox proportional hazards models were adjusted for the following covariates: week of SARS-CoV-2 infection or COVID-19 diagnosis, whether an outpatient visit occurred at the time of testing positive or COVID-19 diagnosis (yes/no),^8^ age group (18‒49, 50‒64, 65‒74, 75‒84, and ≥85 years), sex (male or female), race (Black or African American, White, and other), ethnicity (Hispanic or Latino and not Hispanic or Latino), region (Midwest, Northeast, West, and South), socioeconomic indicators (measured by the area deprivation index [ADI] grouped into quintiles from least to most deprived),^18^ body mass index category (underweight [<18.5], normal weight [18.5‒24.9], overweight [25.0‒29.9], obese [≥30.0], or missing),^19^ Charlson comorbidity index (0, 1, 2, 3, and ≥4), history of medical conditions (yes/no; modeled individually) including cancer, asthma, chronic obstructive pulmonary disease, hypertension, congestive heart failure, atherosclerosis or other heart disease, cognitive disorders including dementia, diabetes, HIV/AIDS, or liver disease, history of immunocompromising conditions or use of immunosuppressive treatment (yes/no),^20^ smoking status (current or former, never, and unknown), prior healthcare interactions (primary care visit in the previous year [yes/no], hospitalization in the previous year [yes/no], emergency department or urgent care visit in the previous year [yes/no]), use of medications with the potential for drug-drug interactions with NMV/r in the 90 days prior to index (use an alternate COVID-19 therapy, hold drug, dose adjust drug, monitor drug, or not on concomitant medication with DDI potential),^16,21^ antibiotic use in the 30 days prior to index (yes/no), COVID-19 vaccination status (up-to-date [defined as receiving at least one dose of XBB vaccine if the index date occurred between September 25, 2023 and March 31, 2024, at least one dose of a BA.4/5 bivalent vaccine if the index date occurred between September 1, 2022 and September 24, 2023, or at least three doses of wild-type COVID-19 vaccine if the index date occurred between April 1, 2022 and August 31, 2022], previously vaccinated against COVID-19 but not up-to-date, and never received COVID-19 vaccine), receipt of influenza vaccine in the previous year (yes/no), receipt of pneumococcal vaccine in the previous 5 years (yes/no), prior documented SARS-CoV-2 infection (yes/no), current (index date to 7 days post index) urinary tract infection (time-varying), history of urinary tract infection in the 30 days prior to index (yes/no), and history of acute respiratory infection in the 30 days prior to index (yes/no).
